# Self-Regulation of the Posterior–Frontal Brain Activity with Real-Time fMRI Neurofeedback to Influence Perceptual Discrimination

**DOI:** 10.3390/brainsci14070713

**Published:** 2024-07-16

**Authors:** Sunjung Kim, Josue Luiz Dalboni da Rocha, Niels Birbaumer, Ranganatha Sitaram

**Affiliations:** 1 Institute of Medical Psychology and Behavioral Neurobiology, University of Tuebingen, 72076 Tuebingen, Germany; 2 St. Jude Children’s Research Hospital, Memphis, TN 38111, USA; josueluiz.dalbonidarocha@stjude.org (J.L.D.d.R.)

**Keywords:** non-conscious and conscious perception, perceptual discrimination, real-time fMRI, neurofeedback, manipulation of perception

## Abstract

The Global Neuronal Workspace (GNW) hypothesis states that the visual percept is available to conscious awareness only if recurrent long-distance interactions among distributed brain regions activate neural circuitry extending from the posterior areas to prefrontal regions above a certain excitation threshold. To directly test this hypothesis, we trained 14 human participants to increase blood oxygenation level-dependent (BOLD) signals with real-time functional magnetic resonance imaging (rtfMRI)-based neurofeedback simultaneously in four specific regions of the occipital, temporal, insular and prefrontal parts of the brain. Specifically, we hypothesized that the up-regulation of the mean BOLD activity in the posterior–frontal brain regions lowers the perceptual threshold for visual stimuli, while down-regulation raises the threshold. Our results showed that participants could perform up-regulation (Wilcoxon test, session 1: *p* = 0.022; session 4: *p* = 0.041) of the posterior–frontal brain activity, but not down-regulation. Furthermore, the up-regulation training led to a significant reduction in the visual perceptual threshold, but no substantial change in perceptual threshold was observed after the down-regulation training. These findings show that the up-regulation of the posterior–frontal regions improves the perceptual discrimination of the stimuli. However, further questions as to whether the posterior–frontal regions can be down-regulated at all, and whether down-regulation raises the perceptual threshold, remain unanswered.

## 1. Introduction

In the long tradition of philosophical and scientific investigations of preconscious processing, the core assumption was that conscious and non-conscious processing was independent of voluntary control mechanisms [1]. The Global Workspace Theory (GWT) is a cognitive theory proposed by Bernard Baars [2]. In Baars’ model, the “global workspace” is a metaphorical space within the brain where information from various cognitive processes is brought together and made available to multiple systems. This global workspace serves as a central hub for integrating information from different sensory modalities, memory systems, and cognitive processes. In the GWT framework, unconscious processes occur in specialized modules, while conscious processes involve the global workspace, where information becomes accessible to multiple cognitive systems [3].

The GNW model of consciousness proposed that modular brain networks are active in parallel and process information unconsciously until neural firing becomes amplified into a self-sustained activity encompassing many distributed regions of the brain [4]. Research has shown that non-conscious responses to invisible stimuli can be observed in sensory cortices in the first 250 ms following stimulus presentation and induce neural activity over longer time periods or larger distances in the brain for eliciting conscious experience [5]. The conscious processing of visual stimuli is accompanied by sustained prefrontal activity, increases in phase synchrony between distant brain regions, enhanced causal interactions between sensory and prefrontal cortices, and an increased complexity of oscillatory interactions within this posterior–frontal network [6]. Recent studies have explored the potential of neurofeedback in enhancing conscious perception. Scharnowski [7] demonstrated that training ongoing spontaneous activity in specific regions of the visual cortex can improve perceptual sensitivity. Vatrano [8] discussed the potential of neurofeedback in treating disorders of consciousness, highlighting its therapeutic applications.

A competing theory of GNW is the recurrent processing theory (RPT), which states that unconscious functions are mediated by a feedforward sweep of neural activity, whereas conscious functions are mediated by recurrent cortico-cortical connections [9]. The prefrontal cortex is involved in providing feedback signals that modulate and shape these recurrent perceptual processes [10]. According to the classic model proposed by Norman and Shallice [11], consciousness arises from the whole-brain orchestration conducted by the prefrontal cortices, which supervise the attentional regulation of lower-level sensorimotor chains [12]. Frontal executive areas, such as the prefrontal cortex, are pivotal in biasing influences on the task-relevant visual cortex [13].

In terms of conscious access to visual information, the GNW theory predicts that there is a first stage of non-conscious processing in the primary visual cortex, followed by a “feed-forward” sweep [14] along the ventral visual pathway in the direction of the frontal regions of the brain, lasting 200–300 ms. Then, in the second stage of processing, the anterior-to-posterior attention network amplifies the visual representation, leading to the conscious perception of the stimulus.

Considering the GNW theory, we asked the following questions: (1) Could brain responses to subliminal emotional stimuli be modulated by the self-control of the posterior–frontal brain activity, and (2) would such a voluntary or self-regulated modulation influence the threshold for conscious perception? To answer these questions, we developed a real-time functional magnetic resonance imaging (rtfMRI) neurofeedback system [15,16,17,18,19] for the instrumental conditioning of BOLD activity in the four regions of interest (ROIs), namely, the right primary visual cortex (PVC; involved in the processing of shape, color and other basic attributes of visual stimuli) [20,21,22], right fusiform face area (FFA; specialized in processing facial stimuli) [23,24,25], left anterior insula (INS; implicated in emotion processing) [26,27] and bilateral anterior medial prefrontal cortex (AMPFC; part of the hypothesized global workspace to sustain the recurrent activation) [3,28,29].

The rtfMRI enables the continuous monitoring of brain activity while individuals receive feedback during real-time task performance. Notably, rtfMRI holds promise for neurofeedback applications, allowing subjects to modulate their brain activity consciously based on immediate feedback. This capability has significant implications for both basic neuroscience research and clinical interventions, offering opportunities to better understand dynamic brain function and develop novel therapeutic approaches for neurological disorders [15].

The selection of the prefrontal cortex (PFC) as a region of interest (ROI) in this study, within the context of the GNW theory, was motivated by the GNW theoretical framework and the role attributed to the PFC in conscious perception. The GNW theory proposes that conscious perception arises from information integration across widespread brain regions, and the PFC is considered a critical hub for this integration. It serves as a central node in the proposed global workspace, orchestrating the flow of information between sensory and higher-order cognitive regions. The PFC is known for its involvement in working memory and cognitive control processes, contributing to the maintenance and manipulation of information. According to GNW, information that reaches the PFC becomes globally accessible, enabling its integration into conscious awareness. Our study aimed to explore how the modulation of the PFC’s activity through neurofeedback might impact conscious perception. The PFC plays a crucial role in executive functions and decision-making processes. The GNW theory suggests that the PFC’s involvement in decision-making contributes to selecting relevant information for conscious access. By targeting the PFC as an ROI, we aimed to investigate whether alterations in its activity could influence perceptual thresholds for emotional face stimuli. Emotional processing, particularly in face recognition, involves interactions between posterior visual regions and the PFC. The fusiform face area (FFA) and insula, included in our posterior–frontal network, are also implicated in emotional face processing. By incorporating the PFC, we sought to capture the broader network involved in emotional perception, consistent with the principles of GNW.

While GNW is a non-localizationist theory, acknowledging the distributed nature of conscious processing, our study employed specific ROIs to investigate how targeted modulation within this network, including the PFC, might impact conscious perception. The PFC’s inclusion aimed to provide insights into the proposed long-distance interactions and the role of higher-order cognitive processes in shaping conscious awareness.

The self-regulation of the BOLD response could be categorized in 2 types: a self-controlled BOLD increase (up-regulation) and a self-controlled BOLD decrease (down-regulation). We hypothesized that the learned up-regulation of the BOLD response in the four ROIs would facilitate conscious access to emotional face stimuli and would reduce the threshold for conscious visual perception of such stimuli. By corollary, we also hypothesized that the learned down-regulation (de-activation) of the four ROIs would raise the threshold for conscious visual perception. Furthermore, the up- and down-regulation conditions were expected to act as mutual control conditions to account for two potential confounds: 1. The modulatory effect of attention in changing the BOLD signal in the ROIs and the processing of the subliminal stimuli; and 2. The effect of perceptual learning (in addition to the hypothesized neurofeedback effects) due to the repeated presentation of the subliminal stimuli during feedback training. The modulatory effect of attention can be compared between the conditions and control by ensuring that the participants are instructed to maintain similar attention levels during the up- and down-regulation conditions. By presenting an equal number of trials of the subliminal stimuli in the up- and down-regulation conditions, the potential effect of perceptual learning in lowering the subliminal threshold would be equalized between the conditions, thus allowing us to compare only the effect of neurofeedback training.

## 2. Materials and Methods

### 2.1. Participants

Fourteen healthy right-handed participants (mean age, 24 years; age range, 21–31; 9 females and 5 males) participated in the study. None of them had any psychiatric or neurological disease and had normal or corrected-to-normal vision. All experimental protocols were approved by the ethical committee (Faculty of Medicine, Tuebingen), and participants signed informed consent forms. All methods were performed in accordance with the relevant guidelines and regulations.

Participants were recruited by an announcement made at the University of Tuebingen for participation in an experiment on “emotion perception using functional magnetic resonance imaging (fMRI)”. Participants were informed that they would receive EUR 10 (USD 13.5) per hour as basic payment for participation in the study. Each experiment consisted of approximately two hours a day for three consecutive days, always at the same time of the day. Each participant was paid EUR 60 (USD 81) for the up-regulation experiment and EUR 60 (USD 81) for the down-regulation experiment; in total EUR 120 (USD 162) as the basic payment for their participation in the study. In addition, participants received a monetary reward proportional to their performance in the rtfMRI training experiments. Participants could earn a maximum of EUR 24 (USD 32.4) per rtfMRI training session, thus totaling EUR 96 (USD 129.6) per rtfMRI training for up-regulation and rtfMRI training for down-regulation. Participants were instructed to maximize their earnings of monetary rewards, presented on the screen after each up/down-regulation trial.

### 2.2. General Methods

**Figure 1:**
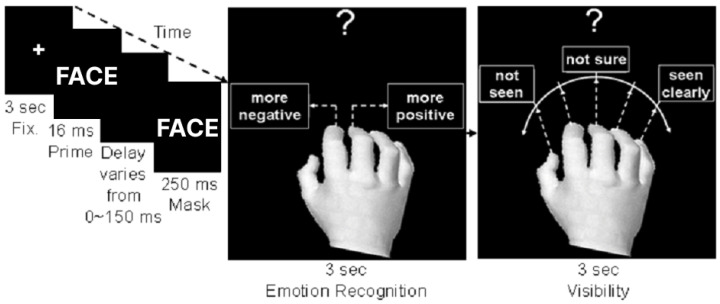
Backward masking task procedure for the pre-test and post-test for the threshold measurement.

**Figure 2:**
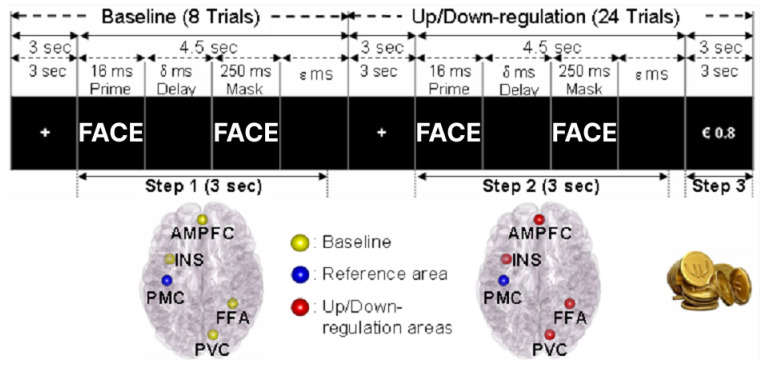
RtfMRI training procedure for up- and down-regulation of posterior–frontal areas. Δ ms is an individual delay per participant (see Materials and Methods). Ε ms is the duration of the presentation of the black screen (see Materials and Methods). Four regions of interest (4ROIs) for up- and down-regulation: PVC = primary visual cortex, FFA = fusiform face area, INS = anterior insula and AMPFC = anterior medial prefrontal cortex. Reference region: PMC = primary motor cortex.

The study was composed of an up-regulation (UR) experiment and a down-regulation (DR) experiment, in random order, whereby the second experiment was performed at least four weeks after the first experiment in order to avoid a cross-over effect. Each experiment consisted of three stages: (i) pretest (on the first day) for measurement of perceptual threshold for detecting emotional faces presented in a backward masking paradigm [28] (Figure 1), (ii) rtfMRI training (on the second and third days) of the four ROIs (Figure 2) and (iii) posttest (on the third day) for the measurement of perceptual threshold after the rtfMRI training (Figure 1). After the end of UR and DR experiments, participants were asked to report the mental strategy they employed for up- and down-regulation and for increasing their monetary reward during rtfMRI training. They were also asked to report how their performance during training related to the monetary reward they received (reward contingency). Participants, in general, reported that they tried to attend to the stimuli as per the experimental instructions. Apart from this, participants did not report any systematic mental strategy for increasing reward.

### 2.3. Pretest and Posttest

The pretest was performed two times in this study: one before and after rtfMRI training in the up-regulation experiment, and one before and after the down-regulation experiment.

Figure 1 shows the procedure of one trial in the pretest and posttest for the measurement of perceptual threshold. In the pretest and posttest, the perceptual threshold of each participant was determined by presenting 192 trials of backward masked stimuli [28] consisting of an emotionally positive or negative face (termed the prime) for 16 ms, followed by a blank screen (termed the delay) with a variable interval (0, 16, 33, 50, 66, 83, 100 and 150 ms), in turn followed by an emotionally neutral face (termed the mask) for 250 ms. After the stimulus presentation, the participant performed an emotion recognition task for 3 sec to report if the prime was more positive or more negative than the neutral mask. Next, the participant performed a visibility task for 3 sec to report the extent to which the prime was clearly seen. The visibility rating included a 5-point Likert scale that ranged from “not seen” to “seen clearly”.

The pretest and posttest were divided into three sessions, to prevent participants from losing focus and concentration on their tasks. Each measurement took 10 min, followed by a 1 min break. Each session consisted of 64 trials; 8 trials for each delay, displayed in randomized order. For each participant, four different faces among sixteen available faces were randomly selected and were presented for this experiment, so that the selected faces consisted of two female and two male faces. The stimuli in each session were composed of 64 pairs of prime and mask, so that the face identities for the prime and the mask were always the same. The color face stimuli were taken from the NimStim Face Stimulus Set [30]. The stimuli were presented using a Pascal based program under Dos 6.2, named Muster5, and were synchronized with a vertical refresh rate (60 Hz) of a screen using a JVC DLA-SX21 projector (JVCKenwood USA Corporations, Long Beach, CA, USA).

### 2.4. Perceptual Threshold

The threshold for conscious perception is calculated using the Weibull function (Kingdom and Prins, 2010 [31]). The Weibull function operationalizes conscious perception based on objective criteria, providing a clear and quantifiable measure. The choice of the Weibull function’s inflection point as a measure of conscious perception is rooted in its utility for capturing the point at which a participant transitions from subliminal to conscious perception. The Weibull function is commonly used in psychophysics to model psychometric curves. It effectively describes the transition between different perceptual states. The inflection point, representing the steepest part of the curve, signifies the threshold at which perceptual performance shifts significantly. Conscious perception is often considered a non-linear transition from unawareness to awareness. With its flexibility in capturing non-linear trends, the Weibull function is well-suited to modeling such transitions. The inflection point corresponds to the stimulus intensity at which participants are most sensitive to changes in conscious perception. It captures the point at which the stimulus becomes more reliably detected, providing a quantitative measure of perceptual awareness. The inflection point of the Weibull function aligns with conscious perception based on the idea that it represents the intensity at which stimuli are reliably discriminated, signifying awareness. The inflection point is often chosen where performance significantly deviates from chance level, indicating a shift from random guessing to systematic discrimination. This aligns with the idea that conscious perception involves reliable discrimination.

To estimate the individual perceptual threshold between conscious and unconscious perception, the data of emotion recognition were fitted with a Weibull psychometric function (www.palamedestoolbox.org) (Kingdom and Prins, 2010 [31]) of the following equation:

(1) $$\begin{array}{l} \Psi (x;\alpha ,\beta ,\gamma ,\lambda )=\gamma +(1-\gamma -\lambda )F_{\omega }(x;\alpha ,\beta ),\\ F_{\omega }(x;\alpha ,\beta )=(1-\exp(-{\left(\frac{x}{\alpha }\right)}^{\beta })), \end{array}$$

where $F_{\omega }(x;\alpha ,\beta )$ is the Weibull function with parameter α determining the x value at which the function reaches its inflection point, β=  the slope of the function, γ=  the guess rate and λ=  the lapse rate. The parameters α, β and λ were estimated using the ‘Palamedes’ toolbox (version 1.1) of the Matlab (version 6.5) routine (http://www.palamedestoolbox.org) [31].

The x value of the inflection point in this function was interpreted as the individual perceptual threshold (TH) for access to consciousness. The individual perceptual threshold (TH) was rounded-down to the nearest value based on the vertical synchronization of the display with 60 Hz refresh rate. This rounded value was called the individual delay (δ). This delay (δ) represents the interval between the presentation of the prime and the mask. In the subsequent up-regulation or down-regulation training experiment, each participant was presented with multiple trials of the backward masking task with this individual delay.

### 2.5. RtfMRI Training

Before the rtfMRI training, participants were instructed to attend to all trials and stimuli equally, irrespective of the type of the experiment (up-regulation or down-regulation) and earn as many rewards as possible. However, participants were not given any information as to whether they were performing up-regulation or down-regulation. Participants were not instructed how to obtain their rewards and were allowed to find their own strategy to obtain rewards. As the study was conceived as an instrumental conditioning paradigm based on contingent reward, participants were informed neither about the aims and hypotheses of the study nor strategies for regulating their brain activity. The purpose of the study was described to the participants as “an experiment to measure their capacity to recognize emotions”.

Participants were required to up or down-regulate dependent on the type of regulation experiment (UR or DR) the four target ROIs, namely, primary visual cortex (PVC), fusiform face area (FFA), anterior insula (INS) and anterior medial prefrontal cortex (AMPFC). The left primary motor cortex (PMC) was used as a reference ROI, to exclude the influence of movement and unspecific arousal on the BOLD signals of the target ROIs. Brain regions were labeled following the Automated Anatomical Labeling (AAL) atlas [32].

RtfMRI training was performed to examine if subjects can learn to up- and down-regulate the posterior–frontal network. The decision to have the same participants perform both up- and down-regulation experiments was based on the need for within-subject comparisons to control individual differences. Using the same set of participants for both conditions allowed us to minimize variability related to factors such as individual cognitive differences, learning abilities, and overall responsiveness to the neurofeedback training.

Additionally, employing the same participants in both conditions enhances the internal validity of the study by ensuring that any observed effects are more likely attributed to the specific manipulation (up- or down-regulation) rather than individual differences between separate groups of participants. This design choice aimed to provide a more robust and controlled assessment of the impact of neurofeedback on conscious perception by directly comparing the effects within each participant across different regulation conditions.

The training protocol was composed of four sessions, whereby two sessions were performed on the second day and two more sessions on the third day. Each session consisted of alternating baseline and up-regulation conditions in the up-regulation experiment, and alternating baseline and down-regulation conditions in the down-regulation experiment (Figure 2).

A visual backward masking task, like the pretest, was combined with the neurofeedback task. Four different faces from the NimStim Face Stimulus Set [30] were used as stimuli. The timing of the stimulus was synchronized with the scanner, so that backward masking and monetary reward presentation were always carried out at the beginning of each repetition time (TR) of the functional imaging pulse sequence. The baseline was composed of eight trials (step 1 in Figure 2), such that in each trial one set of visual stimuli (namely prime, delay and mask) was presented after a fixation cue of 3 sec. In the backward masking task, the visual stimuli were presented in a sequence: (1) a picture of a positive or negative face (prime) for 16 ms, (2) a black screen (delay) for δ ms, previously determined for each participant during the pretest, (3) a neutral face (mask) for 250 ms, and (4) a black screen for ε ms for synchronizing the presentation of trials with the fMRI pulse sequence. The delay (δ) served to make the prime be perceived by the subject subliminally, depending on his/her perceptual threshold. The duration of the black screen (ε) was defined by Equation (2):

(2) ε=4500(ms)−(16(ms)+δ(ms)+250(ms)).

The average of the baseline BOLD levels (BL*_i_*) of the five ROIs in each trial during the 3 sec duration from the onset of the prime was measured (step 1 in Figure 2). These 3 sec contain 2 measurements with TRs of 1.5 sec, defined as j = (1, 2]. After eight trials of baseline, the maximum absolute deviation (MAD) of baseline BOLD values in a set of BL*_i_* was calculated. The MAD was used as a point of reference for each individual to determine the amount of training. MAD is defined by Equation (3):

(3) $$\begin{array}{l} MAD=\max(BL_{i})-\bar{BL},\;\;i=1,\ldots ,8,\\ \bar{BL}=\frac{1}{8}\sum_{i=1}^{8}(BL_{i}),\\ BL_{i}=\frac{1}{2}\sum_{j=1}^{2}(\mathrm{ROI}1_{ij}+\mathrm{ROI}2_{ij}+\mathrm{ROI}3_{ij}+\mathrm{ROI}4_{ij}-\mathrm{ROI}5_{ij}),\;\;i=1,\ldots ,8, \end{array}$$

where ROI1 = BL of the right primary visual cortex (PVC) with 6 voxels, ROI2 = BL of the right fusiform face area (FFA) with 6 voxels, ROI3 = BL of the left anterior insular (INS) with 6 voxels, ROI4 = BL of the bilateral anterior medial prefrontal cortex (AMPFC) with 10 voxels and ROI5 = BL of the left primary motor cortex (PMC) with 6 voxels.

During the up- and down-regulation, participants were presented with backward masking stimuli similar to those in the baseline (step 2 in Figure 2). However, in contrast to the baseline, at the end of each trial of the up- and down-regulation, participants were given visual feedback of the amount of money earned in proportion to the increase in the BOLD signals in the ROIs. The above-described backward masking procedure followed by monetary reward was repeated 24 times for each session of the experiment. In each trial of up- or down-regulation, the difference in the BOLD signal (BOLDDiff) between up- or down-regulation and the baseline for the selected 5 ROIs were computed by Equation (4), whereby the BOLD signal changes during the 3 sec (2 TRs, defined as i = (1, 2]) from the onset of the prime were used as follows for computing the monetary reward:

(4) $$BOLDDiff\;=\frac{1}{2}\sum_{i=1}^{2}(\mathrm{ROI}1_{i}+\mathrm{ROI}2_{i}+\mathrm{ROI}3_{i}+\mathrm{ROI}4_{i}-\mathrm{ROI}5_{i})-\bar{BL}.\;$$

The ratio of up- and down-regulation to baseline was computed as the ratio of BOLDDiff to MAD and was approximated to the closest integer that is greater than the ratio. This approximated value (RewardRate) is defined by Equation (5):

(5) Re⁡wardRate=ceil(BOLDDiffMAD).

RewardRate was an intermediate measure used in the computation of monetary reward. The range of monetary reward was between 0 and 1 in EUR, and the unit of reward was EUR 0.1 (USD 0.135). The monetary reward for up-regulation was computed using a step function defined by Equation (6), while the monetary reward for down-regulation was defined by Equation (7):

(6) Re⁡wardup=0, if Re⁡wardRate≤0;0.1∗Re⁡wardRate, if 0<Re⁡wardRate≤9;1, otherwise. 

(7) Re⁡warddown=0, if Re⁡wardRate≥0;−0.1∗Re⁡wardRate, if −9≤Re⁡wardRate<0;1, otherwise.

The same participants were trained at least four weeks after the up-regulation experiment to down-regulate the four ROIs in a control experiment, in random order, to examine whether changes in perceptual threshold were caused by reward contingency and not by repeated exposure to the stimuli.

### 2.6. FMRI Data Acquisition

Functional images were obtained on a Siemens Trio 3-Tesla MR system (Siemens, Erlangen, Germany) with a standard 12-channel head coil. Sixteen slices (voxel size = 3.3 × 3.3 × 5.0 mm^3^, slice gap = 1 mm) were acquired using a standard echo-planar imaging (EPI) sequence (TR = 1.5 sec, TE = 30 ms, FOV = 210 mm, flip angle α = 70°, matrix size = 64 × 64). For co-registration, a high-resolution T1-weighted structural scan of the whole brain was acquired (MPRAGE, matrix size = 256 × 256, 160 partitions, voxel size = 1.0 × 1.0 × 1.1 mm, TR = 2300 ms, TE = 2.98 ms, TI = 900 ms, FOV = 256 mm, flip angle α = 9°). In order to avoid unnecessary movement unrelated to the experiment, foam cushions between fMRI head coil and both ears of the participant were shunted. Participants were also instructed not to move during the experiment, and to breathe regularly.

### 2.7. RtfMRI Data Acquisition

RtfMRI processing was based upon on the system developed at the Institute of Medical Psychology and Behavioral Neurobiology, Tuebingen [16,17]. The five ROIs were selected by using Turbo Brain-Voyager [version 23.0] (TBV, Brain Innovation, Maastricht, The Netherlands) software [33], so that they comprised rectangular areas of 5 mm thickness (PVC, FFA, INS and PMC with 6 voxels, AMPFC with 10 voxels) from a single slice. Brain regions were labeled following the AAL atlas [32].

### 2.8. Offline fMRI Analysis

**Figure 3:**
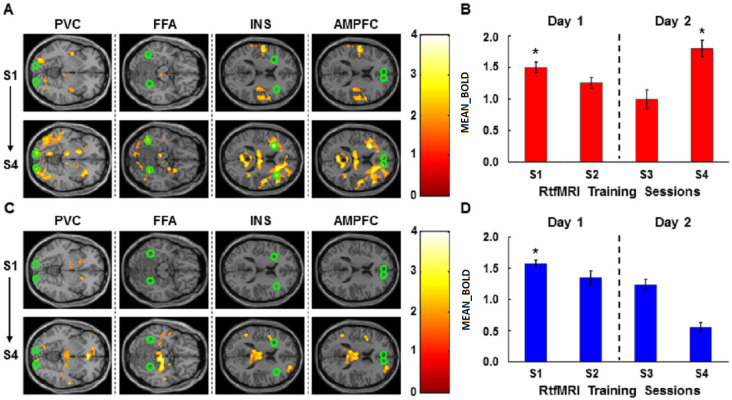
Up- and down-regulation of posterior–frontal areas with rtfMRI training. (**A**) Change in activation levels in the four ROIs (green circles) during up-regulation from rtfMRI training session 1 (S1) to session 4 (S4) (see Materials and Methods). (**B**) Aggregate mean BOLD level in the four ROIs (MEAN_BOLD) in the up-regulation condition in comparison to the baseline condition across training sessions. (**C**) Change in activation levels in the four ROIs (green circles) during down-regulation from rtfMRI training S1 to S4 (see Materials and Methods). (**D**) MEAN_BOLD between down-regulation and baseline across training sessions. For detailed information about MEAN_BOLD in (**B**,**D**), see Materials and Methods. The color bars in (**A**,**C**) indicate T values. Data in B, D were analyzed with Wilcoxon test (n = 14). MEAN_BOLD, mean BOLD change in the four ROIs between up/down-regulation and baseline. * *p* < 0.05.

Offline fMRI analysis was performed to examine the change in activation levels in the four ROIs, across the four feedback training sessions, during up- and down-regulation (Figure 3A,C; Table 1).

Whole-brain analyses were carried out with the Statistical Parametric Mapping software package (SPM8, Wellcome Department of Imaging Neuroscience, London, UK). The functional echo planar imaging volumes of each participant were realigned, co-registered to a T1 image and normalized into the Montreal Neurological Institute (MNI) space by using the anatomical images. These normalized images were smoothed with an 8 mm full-width-half-maximum (FWHW) Gaussian Kernel. A high-pass filter of 1/128 Hz was used for removal of low-frequency noise, and an AR(1) + white noise model was used for temporal autocorrelation.

For a first-level analysis, there were five different types of conditions (FixationBaseline, Baseline, FixationRegulation, Regulation, Reward) in each session. As shown in Figure 2, the condition of “FixationBaseline” denoted the fixation at the start of the baseline condition, while the condition of FixationRegulation meant the fixation at the start of the regulation condition. The condition of “Baseline” denoted the baseline at the start of the backward masking task, while the condition of “Regulation” represented the regulation condition during the backward masking trial. The condition of Reward denoted the presentation of monetary reward at the end of the backward masking trial. The contrast images (Regulation(i) > Baseline(i)) were created session by session for each participant, where i = the number of sessions. These images were then used for a second-level analysis, and the results are reported for statistical thresholds of *p* < 0.05, uncorrected and cluster size k ≥ 5 (Figure 3A,C; Table 1). Brain regions were labeled anatomically following the AAL [32]. The offline fMRI results (Figure 3A,C) are illustrated here using xjView (http://www.alivelearn.net/xjview8/, accessed on 1 March 2008).

### 2.9. RtfMRI Online Analysis

For the up- and down-regulation of the target ROIs, the BOLD values of the selected five ROIs during feedback training (right PVC (ROI1), right FFA (ROI2), left INS (ROI3) and left PMC (ROI5) with 6 voxels and bilateral AMPFC (ROI4) with 10 voxels were extracted using Turbo Brain-Voyager [version 23.0] (TBV, Brain Innovations, Maastricht, The Netherlands) [33].

For ROI analysis of the BOLD signals, the average value of BOLD in the four target ROIs ($\bar{BV_{i}}$) during the 1.5 sec from the onset of prime in the “Baseline” condition per each session was computed by Equation (8), where i = the number of rtfMRI training sessions and j = the trial number:

(8) $$\bar{BV_{i}}=\frac{1}{8}\sum_{j=1}^{8}(\frac{1}{4}(\mathrm{ROI}1_{ij}+\mathrm{ROI}2_{ij}+\mathrm{ROI}3_{ij}+\mathrm{ROI}4_{ij})),\;i=1,\ldots ,4.$$

To estimate the mean BOLD change in four ROIs per session (MEAN_BOLD*_i_*) in the up- or down-regulation experiment, the mean difference in BOLD between baseline and up- or down-regulation conditions, during the 1.5 sec from the onset of the prime, was calculated by Equation (9) (Figure 3B,D), where i = the number of rtfMRI training sessions and j = the number of the trial:

(9) $$MBC\_4ROIs_{i}=\frac{1}{24}\sum_{j=1}^{24}\frac{1}{4}(\mathrm{ROI}1_{ij}+\mathrm{ROI}2_{ij}+\mathrm{ROI}3_{ij}+\mathrm{ROI}4_{ij})-\bar{BV_{i}},\;i=1,\ldots ,4.$$

To estimate mean BOLD change in PMC per session ($MBC\_PMC_{i}$) in the up- and down-regulation experiment, the average value of the BOLD signal in the PMC ($\bar{BVPMC_{i}}$) during the 1.5 sec from the onset of the prime in baseline condition per session was computed by Equation (10), where i = the number of rtfMRI training sessions and j = the number of the trial:

(10) $$\bar{BVPMC_{i}}=\frac{1}{8}\sum_{j=1}^{8}\mathrm{ROI}5_{ij},\;i=1,\ldots ,4.$$

To estimate mean BOLD change in PMC per session ($MBC\_PMC_{i}$) in the up- or down-regulation experiment, the mean difference during the 1.5 sec from the onset of the prime between the baseline and up- or down-regulation conditions was calculated by Equation (11), where *i* = the number of rtfMRI training sessions and j = the number of the trial:

(11) $$MBC\_PMC_{i}=\frac{1}{24}\sum_{j=1}^{24}\mathrm{ROI}5_{ij}-\bar{BVPMC_{i}},\;i=1,\ldots ,4.$$

Besides, we examined mean BOLD change in four ROIs (MEAN_BOLD) during up-regulation in the interval (4.5 sec) between the onset of the prime and the presentation of reward. For this analysis, MEAN_BOLD during the 1.5 sec, 3.0 sec and 4.5 sec from the onset of the prime in each session of up-regulation were computed by Equation (9). This analysis served to examine the relation between the extent of up-regulation of the four ROIs and the change in individual perceptual thresholds.

### 2.10. Analysis of Behavioral Data

In the analysis of behavioral data, trials with no responses were excluded. For the estimation of the individual perceptual threshold in emotion recognition, a Weibull psychometric function was used to fit data of ratio of correct trials across all delays (see the section “Pretest and posttest” in this document and see [31] for more detail).

**Figure 4:**
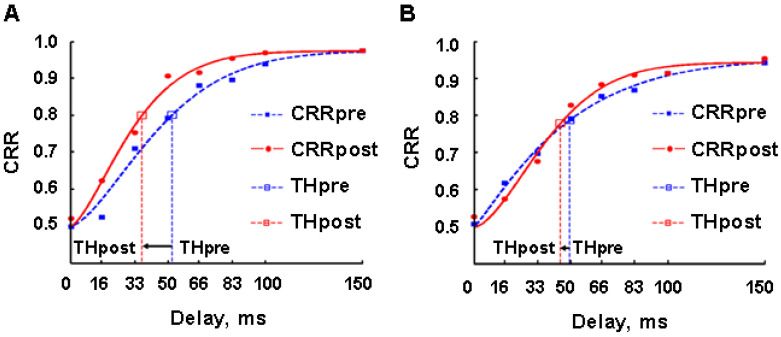
Effects of up- and down-regulation of the posterior–frontal brain activity on perceptual threshold. (**A**) Comparison of perceptual threshold between pretest (tHpre) and posttest (tHpost) in the up-regulation experiment. (**B**) Comparison of perceptual threshold between pretest (tHpre) and posttest (tHpost) in the down-regulation experiment. CRRpre, correct recognition rate in pretest; CRRpost, correct recognition rate in posttest; CRR, correct recognition rate.

**Figure 5:**
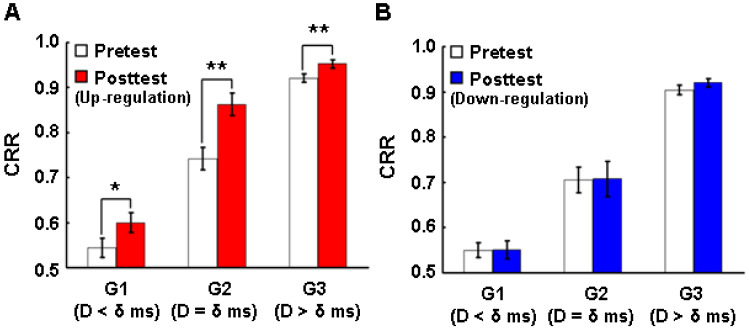
Effects of up- and down-regulation of the posterior–frontal brain activity on correct recognition. (**A**) Comparison of CRR with regard to three different types of stimuli between pretest and posttest in the up-regulation experiment. (**B**) Comparison of CRRs with regard to three different types of stimuli between pretest and posttest in the down-regulation experiment. Data in A and B were analyzed with Wilcoxon test (n = 14). G1, sub-threshold stimuli with the delays below the individual delays (<δ ms); G2, threshold stimuli with the delays at and around the individual delays (=δ ms); G3, conscious stimuli with the delays above the individual delays (>δ ms); CRR, correct recognition rate; D, delay; δ, individual delay (see Materials and Methods). * *p* < 0.05, ** *p* < 0.01.

For emotion recognition, correct recognition rate ($CRR(D_{i})$) in each delay ($D_{i}$) of eight delays (D = {0, 16, 33, 50, 66, 83, 100, 150}) in ms was calculated by Equation (12) (Figure 4A,B and Figure 5A,B), such that NrCorr is the number of correct responses in recognizing the emotional face of the prime and NrResponse is the total number of responses for each delay:

(12) CRR(Di)=NrCorrNrResponse, i=1,…,8.

**Figure 6:**
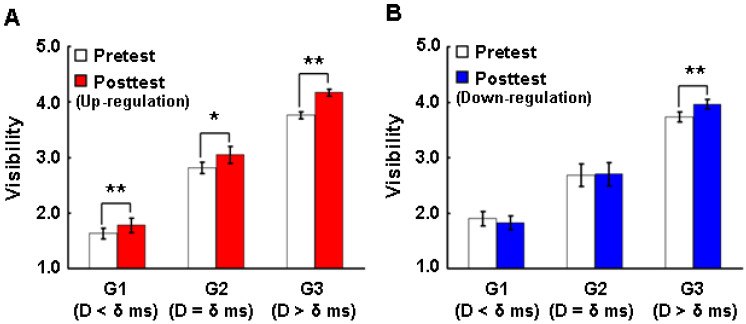
Effects of up- and down-regulation of the posterior–frontal brain activity on visibility. (**A**), Comparison of visibility with regard to three different types of stimuli between pretest and posttest in the up-regulation experiment. (**B**) Comparison of visibility in regard to three different types of stimuli between pre-test and posttest in the down-regulation experiment. Data in A and B were analyzed with Wilcoxon test (n = 14). G1, sub-threshold stimuli with the delays below the individual delays (<δ ms); G2, threshold stimuli with the delays at and around the individual delays (=δ ms); G3, conscious stimuli with the delays above the individual delays (>δ ms); D, delay; δ, individual delay (see Materials and Methods). * *p* < 0.05, ** *p* < 0.01.

For visibility, trials with no responses were excluded. Visibility was rated on a 5-point Likert scale, such that 1 meant “not seen” and 5 meant “clearly seen”. The better the participant saw the prime, the higher the scale of visibility they reported. The visibility (Visibility ($D_{i}$)) in each delay ($D_{i}$) of eight delays (D = {0, 16, 33, 50, 66, 83, 100, 150}) in ms was computed by Equation (13) (Figure 6A,B), where TotalNrRating is the total number of ratings for each delay and VisibilityRating is the actual visibility level reported by the participant.

(13) $$Visibility(D_{i})=\frac{\sum_{j=1}^{TotalNrRating}VisibilityRating_{j}}{TotalNrRating},\;i=1,\ldots ,8.$$

The behavioral data and fMRI data did not obey the Gaussian distribution. Therefore, we performed the Wilcoxon nonparametric test for matched pairs for all statistical analyses.

## 3. Results

### 3.1. Pretest

To ensure that initial differences between experimental conditions did not contaminate our results, we compared the perceptual thresholds in the pretests between the up- and down-regulation experiments, which showed no significant difference (Wilcoxon test, n = 14, Z = −0.722, *p* = 0.470).

### 3.2. RtfMRI Training

The results show that the mean BOLD change in the four ROIs (MEAN_BOLD, see Materials and Methods) between up-regulation and baseline in session 1 (Wilcoxon test, n = 14, Z = −2.291, *p* = 0.022) and in session 4 (Wilcoxon test, n = 14, Z = −2.040, *p* = 0.041) were significantly different from zero (Figure 3B), but not significantly different from zero in sessions 2 and 3. Furthermore, no significant difference in the MEAN_BOLD was found between sessions 1 and 4, although a noticeable increase was observed. Figure 3A and Table 1 present the offline fMRI results comparing up-regulation versus baseline in session 1 and in session 4 across subjects by means of a functional contrast map. The specific and circumscribed activation of the target areas is the desirable outcome of the rtfMRI training. The motor cortex acts as a control area to correct for unspecific and widespread brain activation. We did not observe any significant increase in mean BOLD change in the primary motor cortex, suggesting that there was no unspecific and widespread brain activation. The activation changes in the posterior–frontal ROIs during up-regulation were thus not a consequence of an unspecific BOLD increase due to movement or general brain activation but a consequence of learned self-regulation.

In the down-regulation condition, however, there was no significant reduction in the MEAN_BOLD in any of the sessions. In session 1, MEAN_BOLD was significantly greater in the down-regulation than the baseline (Wilcoxon test, n = 14, Z = −2.919, *p* = 0.004), but was not significantly different from zero in all other sessions (Figure 3C,D). There was no significant difference in mean BOLD change in the primary motor cortex, which was selected as the control brain region.

The above results show that participants could perform only up-regulation but not down-regulation, and that there was no significant learning effect in performing either up- or down-regulation.

### 3.3. Effects of rtfMRI Training on Visual Perception

A posttest of the participants’ perceptual thresholds was performed after up- and down-regulation training of the posterior–frontal brain activity in order to examine the effects of the up- and down-regulation of this network on visual perception. The experimental procedure for the posttest was the same as that of the pretest, except that new face stimuli were used in the posttest to ensure that the regulation of the posterior–frontal brain activity generalizes across different identities of face stimuli (Figure 1).

Each trial consisted of a fixation (Fix), an emotionally positive or negative face (termed the prime) for 16 ms, a blank screen (delay) with a variable interval (0, 16, 33, 50, 66, 83, 100 and 150 ms), ending with an emotionally neutral face (mask) for 250 ms. Subjects reported the facial emotion of the prime compared to mask (emotion recognition), and the visibility of the prime (visibility).

We compared perceptual thresholds after up- and down-regulation. The results show a statistically significant reduction in the perceptual thresholds in the posttest compared to the pretest for the up-regulation experiments (Wilcoxon test, n = 14, Z = −3.296, *p* = 0.001) (Figure 4A). Although there was a slight reduction in the perceptual threshold in the down-regulation condition, no significant reduction in the perceptual thresholds in the posttest compared to the pretest occurred (Wilcoxon test, n = 14, Z = −1.350, *p* = 0.177) (Figure 4B).

The above results show that: (1) significant perceptual threshold changes were observed in the up-regulation experiments (according to our hypothesis) and not in the down-regulation experiments (in contrast to our hypothesis). The above difference in the thresholds between up- and down-regulation conditions indicate that the reduction in the threshold in the up-regulation experiment was not due to repeated exposure to the stimuli. However, the above results do not support our hypothesis that down-regulation is possible, and that it would lead to significant increase in the perceptual threshold.

We examined how conscious and non-conscious perception changes after up- or down-regulation. For this examination, we divided the backward masked stimuli into three different groups of stimuli: sub-threshold stimuli with the delays below the individual delays (see Materials and Methods) (<δ ms) (G1), threshold stimuli with the delays at and around the individual delays (=δ ms) (G2) and conscious stimuli with the delays above the individual delays (>δ ms) (G3). We compared the correct recognition rate (CRR) (see Materials and Methods) belonging to each group between pre- and posttest for up- and down-regulation. The CRRs in the posttest for up-regulation were significantly higher than in the pretest in G1 (Wilcoxon test, n = 14, Z = −2.486, *p* = 0.013), G2 (Wilcoxon test, n = 14, Z = −2.832, *p* = 0.005) and G3 (Wilcoxon test, n = 14, Z = −3.006, *p* = 0.003) (Figure 5A), while there was no significant change in CRR in any of the three groups after down-regulation (Figure 5B). These results indicate that up-regulation improves perceptual discrimination.

We further explored if visibility (see Materials and Methods) is modified with regard to sub-threshold (G1), threshold (G2) and conscious stimuli (G3) after up- and down-regulation. The visibility in the posttest for up-regulation was significantly higher than in the pretest in G1 (Wilcoxon test, n = 14, Z = −2.795, *p* = 0.005), in G2 (Wilcoxon test, n = 14, Z = −2.119, *p* = 0.034) and in G3 (Wilcoxon test, n = 14, Z = −5.740, *p* = 0.000) (Figure 6A). In comparison to the effect of up-regulation on visibility, there was no significant change in visibility for G1 and G2 after down-regulation. In G3, there was a significant positive change in visibility after down-regulation (Wilcoxon test, n = 14, Z = −3.924, *p* = 0.000) (Figure 6B).

These results indicate that the up-regulation of the posterior–frontal brain activity not only reduced the perceptual thresholds of the participants, but also improved their performance in emotion recognition and visibility.

### 3.4. Neural Marker of Perceptual Change

**Figure 7:**
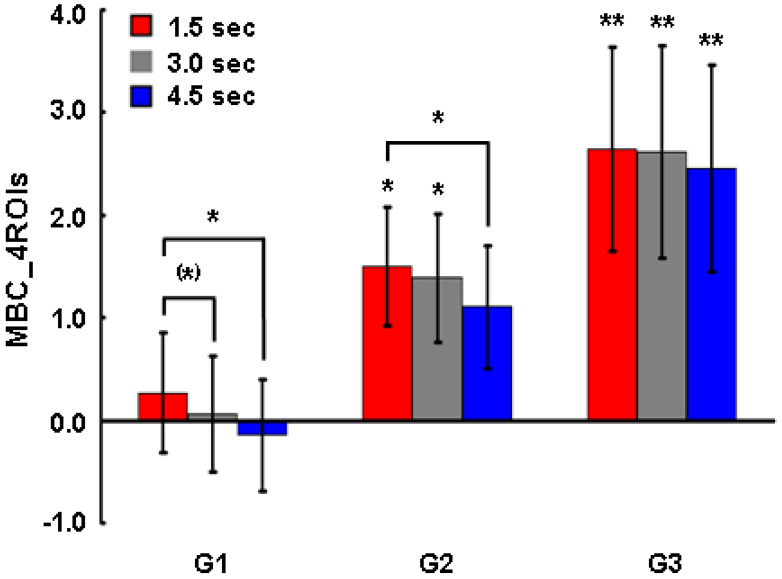
Neural marker of perceptual change. Change in MEAN_BOLD across all four sessions during up-regulation in three different groups: G1 (n = 5) with ∆TH ≤ 10 ms, G2 (n = 5) with 10 ms < ∆TH ≤ 20 ms, G3 (n = 4) with ∆TH > 20 ms in the posttest of the up-regulation experiment. Data were analyzed with Wilcoxon test. MEAN_BOLD, mean BOLD change in the four ROIs between up-regulation and baseline; ∆TH, change in perceptual threshold. (*) *p* < 0.1, * *p* < 0.05, ** *p* < 0.01.

We examined the difference in the MEAN_BOLD between good performers, showing a large change in perceptual threshold, and bad performers, showing a small change in perceptual threshold. We divided the subjects into three groups based on the extent of the change in perceptual threshold (∆TH): Group 1 (G1, n = 5) with ∆TH ≤ 10 ms, Group 2 (G2, n = 5) with 10 ms < ∆TH ≤ 20 ms and Group 3 (G3, n = 4) with ∆TH >20 ms in the posttest of the up-regulation experiment (Figure 7). Bad performers (G1) did not up-regulate significantly across all the four sessions in the three different durations from the onset of prime, but showed a significant difference in MEAN_BOLD not only between 1.5 sec and 3.0 sec (Wilcoxon test, n = 5, Z = −1.904, *p* = 0.057) but also between 1.5 sec and 4.5 sec (Wilcoxon test, n = 5, Z = −2.093, *p* = 0.036) (Figure 7, left). Normal performers (G2) showed significant differences in the MEAN_BOLD from zero to 1.5 sec (Wilcoxon test, n = 5, Z = −2.352, *p* = 0.019), at 3.0 sec (Wilcoxon test, n = 5, Z = −2.016, *p* = 0.044) from the onset of prime, and also a significant difference in MEAN_BOLD between 1.5 sec and 4.5 sec (Wilcoxon test, n = 5, Z = −2.558, *p* = 0.011) (Figure 7, middle). Good performers (G3) up-regulated significantly across all four sessions at 1.5 sec from the onset of the prime (Wilcoxon test, n = 4, Z = −2.896, *p* = 0.004), at 3.0 sec from the onset of the prime (Wilcoxon test, n = 4, Z = −2.689, *p* = 0.007) and at 4.5 sec from the onset of the prime (Wilcoxon test, n = 4, Z = −2.585, *p* = 0.010) (Figure 7, right), and also maintained the extent of up-regulation longer in comparison to the bad performers (G1).

## 4. Discussion

Previous studies have reported that conscious perceptual sensitivity could be improved by means of neurofeedback training of visual areas [7,34,35]. The present study demonstrates for the first time that the up-regulation of the posterior–frontal brain network improves perceptual sensitivity not only for suprathreshold visual stimuli but also for subthreshold visual stimuli.

However, contrary to our hypotheses, participants could only perform the up-regulation of the posterior–frontal regions and consequently lowered their perpetual threshold, but did not achieve the down-regulation of the regions and did not show an increase in perceptual thresholds due to training. The study suggests that up-regulation training of the posterior–frontal regions lowered the threshold of conscious perception. However, our results fall short of establishing if it is physiologically possible for participants to reduce BOLD activation levels in the multiple posterior–frontal regions of the brain below the baseline levels, and if and how down-regulation relates to changes in perceptual threshold.

On the positive side, the down-regulation experiment acted as a direct control to the up-regulation experiment, in being able to account for both the attention-related effects of neurofeedback training and the perceptual learning effects due to repeated presentation of the visual stimuli. One may argue that the participants might have used selective/enhanced attention for up-regulation but not down-regulation. All they were provided was a reward contingent on a desirable increase or decrease in BOLD levels in the ROIs.

Another criticism could be that the reduction in perceptual threshold is not due to the up-regulation of the posterior–frontal regions, but merely due to perceptual learning of the repeatedly presented stimuli. However, this criticism is also not valid as both the up- and down-regulation experiments presented an equal number of backwardly masked stimuli, and, hence, one would expect the same level of perceptual learning in both conditions. In essence, in the absence of any neurofeedback effect and only attentional and perceptual learning effects, both the up- and down-regulation training should have produced identical changes in the threshold. The fact that up-regulation alone produced an increase in the activation levels in the regions, and only up-regulation produced a significant reduction in perceptual threshold, clearly establishes the specific effect of neurofeedback training on up-regulation.

First, the up-regulation of this network caused a significant reduction in perceptual thresholds (Figure 4A), while down-regulation training did not cause a significant reduction in the perceptual threshold (Figure 4B). Considering that the initial perceptual thresholds of the participants before up- and down-regulation were not significantly different, the reduction in the perceptual threshold can be directly attributed to the neurofeedback training. Second, the up-regulation of this network brought a significant improvement in emotion recognition, not only at the conscious perceptual level but also below and above the thresholds (Figure 5A), while the down-regulation of this network did not induce any effect on emotion recognition at any threshold range (Figure 5B). Third, the up-regulation of this network significantly improved the reported visibility of the visual emotional stimuli not only at the conscious thresholds of participants but also below and above the thresholds (Figure 6A), while down-regulation training increased the visibility significantly above the thresholds, although a significant down-regulation of the BOLD signal in the ROIs was not observed (Figure 6B).

No systematic knowledge of reward contingency was reported by the participants, either for up-regulation or for down-regulation [36,37] (see Materials and Methods). Knowledge of the contingency or conscious imagery [16] did not determine the present results. The neural mechanisms of learning the self-regulation of brain activity are comparable to skill learning, previously reported for the neurofeedback of neuroelectric excitation patterns, such as cellular firing [15,38,39] and neuroelectric cortical activity [40]. The strong relationship between the degree of the BOLD up-regulation of the neuronal network of emotional face recognition and the reduction in the perceptual threshold suggests a causal effect of the instrumentally learned physiological regulation on the specific behavior, namely perceptual threshold, and access to consciousness. In addition, the down-regulation results support this conclusion: the down-regulation of BOLD across sessions in the target network was not possible, probably due to the incompatibility of the training task requiring increased effort and activity simultaneously with the required decrease in brain activity in most of these brain regions. But even that effort did not result in an increase in BOLD comparable to up-regulation training, demonstrating that the reward contingency was effective in both conditions, but less so during down-regulation. The down-regulation of the posterior–frontal brain activity did not result in a significant reduction in the mean BOLD change across the four sessions.

Several factors may contribute to the failure of down-regulation. Down-regulation tasks in neurofeedback involve inhibiting or reducing the neural activity in specific brain regions. Participants were required to down-regulate the posterior–frontal network, which may have posed a challenge due to the simultaneous demand for effort and inhibition. The cognitive demand of suppressing neural activity while engaging in the task could be more demanding than the effort-related up-regulation. The down-regulation task might inherently be more challenging than up-regulation. It is possible that the participants found it difficult to exert control over the targeted posterior–frontal regions to reduce their activity consistently across sessions. Participants may have unintentionally developed strategies that were more conducive to up-regulation than down-regulation. The neurofeedback task may have led to non-specific learning, where participants inadvertently focused on aspects unrelated to the intended down-regulation, such as selective attention or emotional processing. The reward contingency associated with neurofeedback may have played a role. Participants might have been more successful in detecting and reinforcing increased neural activity (up-regulation) compared to inhibiting or reducing activity (down-regulation). Exploring alternative reward structures or reinforcing specific aspects of the down-regulation process could be considered. In summary, the failure of down-regulation could be attributed to the inherent complexity of inhibitory tasks, potential non-specific learning, and the challenges associated with balancing effort and inhibition. Although participants were given visual feedback of the amount of money earned in proportion to the increase in the BOLD signals in the ROIs, it is unknown whether it contributed to increase the reliability of the training.

In our methods, we used a list of 13 equations: Equation (1) was applied to estimate the individual perceptual threshold between conscious and unconscious perception; Equation (2) was to determine the duration of the black screen; Equation (3) determined the maximum BOLD deviation across the eight baseline trials; Equation (4) accessed the difference in the BOLD signal between up- or down-regulation and baseline; Equation (5) calculated the ratio of up- and down-regulation to baseline, while Equations (6) and (7) converted these values into monetary rewards. Equation (8) computed the average value of BOLD in the four target ROIs in the baseline condition per each session, while Equation (9) computed the mean BOLD change for the up- or down-regulation. Equations (10) and (11) estimate the mean BOLD change in PMC in the up- and down-regulation experiment. Equation (12) calculates the correct recognition rate. Finally, Equation (13) computed the visibility in each of the eight tested black screen delays.

Our findings may contribute to the general understanding of conscious perception, and particularly to that of emotionally laden visual stimuli. The conscious perception of emotional faces is a complex process involving the registration of visual information, feature selection, face perception, and emotional perception. The PVC of humans is involved in the processing of visual information as the recipient of visual input [41] but its direct influence on conscious perception is still debatable [9,42,43,44]. The FFA is known as a brain region for face perception that responds to faces [23,24,25,45,46], but its degree of participation in emotional perception is controversial [47,48,49,50]. FFA is mainly involved in discriminating among the categories of emotions in faces [47,50], whereas other studies showed that the insula and amygdala are also involved in emotional processing [48,49]. Further, the PFC is critical for perceiving the contents of visual information by holding and manipulating it in working memory [29,51]. Based on the above, we selected the PVC, the FFA, the insula and PFC as the regions of interest, which extend from the posterior to the prefrontal regions of the human brain in line with the global workspace theory [5,6,24,52,53,54].

The RPT provides an additional framework to understand our findings. According to RPT, conscious perception arises from recurrent interactions between higher-order and lower-order visual areas. This study aligns with RPT by demonstrating that the up-regulation of BOLD signals in the posterior–frontal network, including the PVC and FFA, enhances perceptual discrimination. These recurrent feedback loops are crucial in modulating perceptual thresholds, reinforcing the importance of both feedforward and feedback processes in conscious visual perception. However, our work does not test any specific hypothesis for distinguishing between the GNW and RPT. Further work is needed to conduct a direct empirical comparison of the two theories.

The ability to up-regulate posterior–frontal brain activity holds promise for various clinical applications. By enhancing attention, memory, and decision-making, it could aid cognitive deficits and benefit mental health therapies for conditions like depression, anxiety, and post-traumatic stress disorder. Additionally, modulating these regions may offer non-pharmacological interventions for chronic pain management and aid neurorehabilitation efforts following stroke or traumatic brain injury. Neurofeedback training targeting these areas could also accelerate learning processes in education and skill acquisition, while, ethical considerations aside, voluntary modulation might lead to cognitive enhancement, with potential impacts on academic or professional performance. Overall, the potential clinical applications of up-regulating posterior–frontal brain activity are broad, spanning cognitive enhancement, mental health, pain management, neurorehabilitation, learning, and neuroenhancement, though further research is required to optimize its therapeutic efficacy.

## 5. Conclusions

Several lines of evidence in this study indicate that the up-regulation of the posterior–frontal network increases our ability to perceive emotionally laden human face images. Furthermore, our results show that the observed improvement in perceptual ability is a specific effect of rewarding the successful up-regulation of the posterior–frontal brain activity and is not explained by the repeated exposure to the stimuli, general arousal or effort. These findings partially support the GNW hypothesis of consciousness perception, to the extent that the up-regulation of the posterior–frontal regions improved the conscious awareness of stimuli. However, further questions as to whether the posterior–frontal regions can be down-regulated at all, and whether down-regulation raises the perceptual threshold, remain unanswered. The rtfMRI neurofeedback approach used in this study opens the possibility for manipulating the threshold of awareness through physiological learning.

## Figures and Tables

**Table 1:** Activation in the rtfMRI training session 4 during up-regulation.

Brain Region	MNI Coordinates			*p* Value (Uncorrected)	Cluster Size
	x	y	z		
Left hemisphere					
Primary visual cortex	−9	−92	−5	0.021	41
Fusiform face area	−42	−56	−15	0.013	31
Anterior insula	−29	17	10	0.000	55
Anterior medial prefrontal cortex	−5	46	15	0.022	9
Right hemisphere					
Primary visual cortex	21	−92	−5	0.020	13
Fusiform face area	24	−53	−15	0.022	19
Anterior insula	34	20	15	0.009	17
Anterior medial prefrontal cortex	4	46	20	0.001	9

x, y, z = coordinates according to the MNI stereotactic space for clusters in the left and right hemispheres contributing to the rtfMRI training session 4 during up-regulation, peak *p* value (*p* < 0.05, uncorrected), cluster size k ≥ 5.

## Data Availability

The data presented in this study are available on request from the corresponding author due to the institutional guidelines for maintaining anonymity and integrity, and the study investigators’ interest in developing new collaborative opportunities.
